# Association of Herpesvirus and Periodontitis: A Clinical and Laboratorial Case–Control Study

**DOI:** 10.1055/s-0043-1761423

**Published:** 2023-06-09

**Authors:** Marta Picolo, Miguel A. de Araújo Nobre, Francisco Salvado, Helena Barroso

**Affiliations:** 1 Mestrado Integrado de Medicina Dentária, Instituto Universitário Egas Moniz (IUEM), Caparica, Portugal; 2 Clínica Universitária de Estomatologia, Faculdade de Medicina, Universidade de Lisboa, Portugal; 3 Research and Development Department, Maló Clinic, Lisboa, Portugal; 4 Centro de Investigação Interdisciplinar Egas Moniz (CiiEM) – Instituto Universitário Egas Moniz (IUEM), Caparica Portugal

**Keywords:** periodontitis, herpes simplex virus, Epstein-Barr virus, cytomegalovirus, epidemiology

## Abstract

**Objectives** A significant influence of the Herpesviridae family in the progression of periodontal disease has been suggested. The aim of this study was to investigate the potential association of four Herpesviruses (HSV-1, HSV-2, cytomegalovirus [CMV], and Epstein-Barr virus [EBV]) with periodontal disease using a qualitative test for evaluating the presence or absence of viral DNA in crevicular fluid samples of both healthy periodontal patients and periodontal compromised patients.

**Materials and Methods** A case–control study was conducted in 100 participants at a university clinic. A qualitative test was used for evaluating the presence/absence of viral DNA in crevicular fluid samples of both healthy periodontal patients and periodontal compromised patients, and considering the periodontitis staging (stage II, stage III, and stage IV) and grading (grade A, grade B, and grade C).

**Statistical Analysis** The distribution of the same exposure variables to the periodontitis staging and grading was compared using Chi-square, Fisher's exact, and Gamma tests depending on the variable characteristics. The significance level was set at 5%. The association of the variables: age, sex, diabetes, smoking, alcohol, and oral hygiene was also considered.

**Results** The prevalence of Herpesviridae family virus DNA was 6% for the periodontal healthy group and 60% for the periodontitis group (roughly 60% on periodontitis stages II, III, and IV, *p* <0.001; and twofold increase in moderate and rapid progression grades compared with the slow progression grade, *p* <0.001). HSV1 DNA was prevalent in all periodontitis stages and grades. HSV 2, EBV, and CMV DNA had increasing prevalence rates in more severe stages (stages III and IV, *p* <0.001); while considering periodontitis grade, HSV2 ( *p* = 0.001), CMV ( *p* = 0.019) and EBV ( *p* <0.001) DNA were prevalent only in grades B and C, with EBV DNA registering a marked prevalence in grade C.

**Conclusion** A significant different distribution of Herpesviridae virus DNA per each stage of disease was registered.

## Introduction

Periodontitis is defined as a chronic multifactorial inflammation of the periodontium associated with a microbial imbalance, while leading to a progressive destruction of the teeth supporting tissues. Clinically, the pathology manifests with clinical attachment loss, radiographical evidence of alveolar bone loss, presence of periodontal pockets and gingival bleeding. 1 Importantly, periodontitis is the main cause of tooth loss globally. 2 Occurring more frequently around the third decade of life, 3 the prevalence of periodontal disease on most countries surpasses 50% 4 and its worldwide prevalence is estimated to be 11.2%. 5 By culminating in masticatory disability and leading to aesthetical consequences it can severely affect an individual's quality of life by being a determinant of social bias. 1

Periodontitis is multifactorial, with various environmental, systemic, and genetic factors playing a role in its development. 6 Calculus was initially thought to be its only cause, although the theory about its accumulation of biofilm has lost emphasis. 3 7 From an epidemiological point of view, biofilm accumulation alone is not considered a sufficient and necessary cause for periodontitis onset, given the existence of cases of severe plaque accumulation with mild periodontitis and cases of significant periodontium destruction with low biofilm accumulation. 8 Accordingly, treatments focusing on removing dental calculus relate to different outcomes depending on the clinical situation. 9 Periodontitis is marked by periods of remission interrupted by periods of clinical collapse, 3 10 whose biological origin is not clearly explained. Hence, these patients demand a thorough follow-up. 11

The virulence of infectious microorganisms is highly determinant on the progression of the disease. However, environmental, immune, demographic, and viral factors have also been suggested. 10 With the evaluation of disease progression, viruses began to be considered 10 with a spotlight on the Herpesviridae family, 12 where in the 1990s the Herpes Simplex virus (HSV-1, HSV-2), Epstein-Barr virus (EBV), and cytomegalovirus (CMV), were associated with periodontal disease. 12 There might be a potential relationship between herpesvirus and periodontal disease, considering the 67 and 13% worldwide prevalence of HSV1 and HSV2 infections, respectively. 13 In periodontal compromised patients these viruses are found quite frequently reaching large copy counts, 14 whether in samples of gingival tissue, crevicular fluid, or subgingival plaque. 15 Herpesviridae viruses can also act by distinctive mechanisms that could potentially infer their association with periodontitis. Examples of such mechanisms include: causing a decrease in the effectiveness of the host's immune response, facilitating colonization by periodontal pathogenic bacteria, creating antibodies against neutrophils causing secondary infections, 16 or causing tissue damage and altering inflammatory mediators. 12 Both HSV, EBV and CMV cause latent infections, whose periodic reactivation aligns with the most common course pattern of periodontal disease. 17 CMV viruses and EBV lead to an increased expression of IL-1β (interleukin-1β) and TNFα (tissue necrosis factor) by macrophages and monocytes. Consequently, TNFα and IL-1α can stimulate matrix metalloproteinases (MMPs) and decrease inhibitors of these enzymes, inevitably mediating the periodontium's alveolar bone destruction. 16 Considering the literature, a significant influence of the Herpesviridae family in the progression of periodontal disease has been suggested. Nevertheless, studies investigating the association of these virus and the different stages of periodontitis are lacking. The aim of this study was to investigate the potential association of four Herpesviruses (HSV-1, HSV-2, CMV, and EBV) with periodontal disease using a qualitative test for evaluating the presence or absence of viral DNA in crevicular fluid samples of healthy periodontium patients and periodontal compromised patients.

## Materials and Methods

### Study Design and Setting

This case–control clinical study was conducted between March and July 2019 at the Egas Moniz University Dental Clinic (Almada, Portugal). This study was approved by the human subject's ethics board of Cooperativa de Ensino Superior Egas Moniz, CRL (approval n. 708) and conducted in accordance with the Helsinki Declaration of 1975, as revised in 2013. Written informed consent was granted for all subjects. The protocol was registered in the ClinicalTrial.gov Protocol registration and results system (ID pending).

### Participants

Patients (>18 years of age) with natural teeth attending clinical appointments at the Egas Moniz University Dental Clinic represented the population eligible for the study as convenience sample. Cases and controls were selected from the same population. Cases were defined as patients with natural teeth and at least one tooth diagnosed with periodontitis during the first clinical appointment at the department of periodontology. Controls were defined as patients with natural teeth without a diagnose of periodontitis and selected from the remaining clinical specialty departments at the same university clinic in an effort to prevent selection bias. Exclusion criteria were applied for both groups. These comprised the occurrence of a viral infection during 6 months prior to the clinical study, exposure to antiviral medication in the 6 months prior to the survey, clinical history of immunosuppression, either exogenous or endogenous, and chemotherapy in the previous 6 months. A total of five patients (from 105 patients screened at baseline) were excluded due to the presence of the abovementioned criteria.

### Procedures

The periodontal diagnosis was classified according to the staging and grading scheme ( Appendix Tables 1 and 2 ). 1 2 Samples from healthy patients were collected after a thorough examination. Individuals presenting gingival bleeding compatible with gingivitis or evidence of periodontal pockets were excluded. The periodontal diagnosis was, in both cases, assessed through the measurements of probing depth, gingival height (measuring the height of keratinized and attached gingiva), 18 degree of tooth mobility, tooth loss, levels of clinical attachment alongside gingival index, 19 and plaque index. 20 Additionally, it was considered the presence of diabetes and smoking habits in light of their role as periodontitis grade modifiers. Recall bias was judged to be unlikely given the type of variables collected.

Unstimulated crevicular fluid samples were collected using two-sized 35 paper cones per subject, 21 both used in the sampling process. The paper cones were previously sterilized to eliminate microorganisms and increase the absorption potential. 22 Before the sampling procedure, the periodontal pockets were remeasured to confirm their depth and the collection sites were dried with an air–water syringe. Two paper cones were placed, one in each pocket with greater probing depth, for 30 seconds. 23 In healthy periodontium patients, the paper cones were positioned in the gingival sulci of premolars and molars. Subsequently, samples were placed in sterile Eppendorf and assigned a number for future identification.

First, DNA was extracted from each sample and multiplex PCR was carried at the Egas Moniz Applied Microbiology Laboratory (LMAEM). The laboratorial procedures used in the present study followed previously published methodology 24 and is extensively described in appendix.

### Outcome and Exposure Variables

Considering the outcome variables and based on the clinical evaluation, the patients were classified according to their periodontal status (healthy group and periodontally compromised group) and periodontitis staging according to the World workshop on the classification of periodontal and peri-implant diseases and conditions (stages I to IV). 1 2 Considering the exposure variables, the demographic variables collected were sex (female and male) and age, defined as the number of life years and recorded in an ordinal scale ([18–25], [26–45] and ≥46 years). The information on diabetes was collected based on the potential influence on the periodontal outcome. 25 Information on deleterious habits such as smoking (smoker, non-smoker) and alcohol consumption (yes, no) were also collected. 26 27 The oral hygiene habits considering the frequency of daily brushing was collected and categorized (0–1 times per day, 1–2 times per day, and 2–3 times per day). The presence of virus DNA (HSV1, HSV2, EBV, and CMV) was registered dichotomously (present or absent).

### Statistical Analysis

The authors assumed a Herpesviridae infection prevalence rate of 45% in periodontally compromised patients and 7% in periodontally healthy patients, based on the median infection prevalence rates retrieved from studies. 28 The target sample size to detect the hypothesized effect sizes was 50 cases and 50 controls (95% power, 5% significance level, two-sided tests, allowing for a non-completion rate of 35%). Frequencies and correspondent category percentages were computed for the categorical variables. The association between the variables: age, sex, diabetes, smoking, alcohol, oral hygiene, and presence of virus DNA (HSV1, HSV2, EBV, and CMV) and the periodontal status (periodontally compromised and periodontally healthy groups) were computed using Chi-square and Fisher's exact tests. The distribution of the same exposure variables in the periodontitis staging and grading was compared using Chi-square, Fisher's exact, and Gamma tests depending on the variable characteristics. The significance level was set at 5%. The analyses were performed with SPSS 26 (IBM, New York, United States).

This manuscript was written following the STROBE (Strengthening the Reporting of Observational Studies in Epidemiology) guidelines. 29

## Results

### Study Subjects

One hundred participants were included (54 women, 46 men) with a predominance of patients in 26 to 45 years age group, none declined participation. Patient demographics and characteristics are provided in Table 1 . The participants on the healthy periodontal group were significantly younger and with a higher prevalence of women when compared with periodontal compromised group ( Table 1 ). The periodontal compromised group exhibited a higher prevalence of diabetes ( *n* = 2 patients, compared with *n* = 1 patient in the healthy group), smoking habits, alcohol habits, and lower prevalence of oral hygiene brushing habits, nevertheless not statistically significant when compared with the healthy periodontal group.

**Table 1:** Sample characteristics and inferential analysis comparing the distribution of variable categories between cases and controls

	Sample	Periodontal groups		
Variable	Total	Controls	Cases	*p* -Value
Part, *N* (%)	100 (100)	50 (50)	50 (50)	–
Age groups				0.012 a
[18–25]	2 (2)	0 (0)	2 (4)	
[26–45]	61 (61)	39 (78)	22 (44)	
≥46	37 (37)	11 (22)	26 (52)	
Sex				0.045 a
Male	46 (46)	18 (36)	28 (56)	
Female	54 (54)	32 (64)	22 (44)	
Diabetes				1
No	97 (97)	49 (98)	48 (96)	
Yes	3 (3)	1 (2)	2 (4)	
Smoking				0.159
No	55 (55)	31 (62)	24 (48)	
Yes	45 (90)	19 (38)	26 (52)	
Alcohol				0.155
No	59 (59)	33 (66)	26 (52)	
Yes	41 (41)	17 (44)	24 (48)	
Oral hygiene				0.314
0–1 times/d	10 (10)	3 (6)	7 (14)	
1–2 times/d	20 (20)	9 (18)	11 (22)	
2–3 times/d	70 (70)	38 (76)	32 (64)	
Herpesviridae family				<0.001 a
Absence	67 (67)	47 (94)	20 (40)	
Presence	33 (33)	3 (6)	30 (60)	
Virus DNA prevalence				
HSV1				0.001 a
Absence	80 (80)	49 (98)	31 (62)	
Presence	20 (20)	1 (2)	19 (38)	
HSV2				0.001 a
Absence	87 (87)	49 (98)	38 (76)	
Presence	13 (13)	1 (2)	12 (24)	
EBV				<0.001 a
Absence	83 (83)	49 (98)	34 (68)	
Presence	17 (17)	1 (2)	16 (32)	
CMV				0.027 a
Absence	94 (94)	50 (100)	44 (88)	
Presence	6 (6)	0 (0)	6 (12)	

Abbreviations: CMV, cytomegalovirus; EBV, Epstein-Barr virus; HSV, herpesviruses.

Note: Percentages refer to columns.

a Significant difference.

Considering the periodontitis staging, the age distribution was significantly different between groups, with older patients on the more severe stage groups. No significant differences were registered in the distribution of sex, smoking, alcohol consumption, and oral hygiene habits ( Table 2 ). Considering the periodontitis grade of progression, the age and smoking distribution were significantly different between groups, with older patients and smokers on the higher progression groups. No significant differences were registered in the distribution of sex, alcohol consumption and oral hygiene habits ( Table 3 ).

**Table 2:** Sample characteristics and inferential analysis comparing the distribution of variable categories between periodontitis stage

	Periodontitis stage				
Variable	Healthy	Stage II	Stage III	Stage IV	*p* -Value
Part, *N* (%)	50 (50)	5 (5)	32 (32)	13 (13)	–
Age groups					<0.001 ^a^
[18–25]	0 (0)	0 (0)	2 (6.3)	0 (0)	
[26–45]	39 (78)	5 (100)	15 (46.9)	2 (15.4)	
≥46	11 (22)	0 (0)	15 (46.9)	11 (84.6)	
Sex					0.061
Male	18 (36)	1 (20)	22 (68.8)	5 (38.5)	
Female	32 (64)	4 (80)	10 (31.3)	8 (61.5)	
Diabetes					0.244
No	49 (98)	5 (100)	32 (100)	11 (84.6)	
Yes	1 (2)	0 (0)	0 (0)	2 (15.4)	
Smoking					0.275
No	31 (62)	1 (20)	16 (50)	7 (53.8)	
Yes	19 (38)	4 (80)	16 (50)	6 (46.2)	
Alcohol					0.101
No	33 (66)	4 (80)	16 (50)	6 (46.2)	
Yes	17 (34)	1 (20)	16 (50)	7 (53.8)	
Oral hygiene					0.321
0–1 times/d	3 (6)	2 (40)	4 (12.5)	1 (7.7)	
1–2 times/d	9 (18)	1 (20)	8 (25)	2 (15.4)	
2–3 times/d	38 (76)	2 (40)	20 (62.5)	10 (76.9)	
Herpesviridae family					<0.001 ^a^
Absence	47 (94)	2 (40)	13 (40.6)	5 (38.5)	
Presence	3 (6)	3 (60)	19 (59.4)	8 (61.5)	
Virus DNA prevalence					
HSV1					<0.001 ^a^
Absence	49 (98)	3 (60)	19 (59.4)	9 (69.2)	
Presence	1 (2)	2 (40)	13 (40.6)	4 (30.8)	
HSV2					<0.001 ^a^
Absence	49 (98)	4 (80)	26 (81.3)	8 (61.5)	
Presence	1 (2)	1 (20)	6 (18.8)	5 (38.5)	
EBV					<0.001 ^a^
Absence	49 (98)	4 (80)	24 (75)	6 (46.2)	
Presence	1 (2)	1 (20)	8 (25)	7 (53.8)	
CMV					0.047 ^a^
Absence	50 (100)	5 (100)	28 (87.5)	11 (84.6)	
Presence	0 (0)	0 (0)	4 (12.5)	2 (15.4)	

Note: Periodontitis staging and grading 1 2 ; Percentages refer to columns.

* Significant difference.

**Table 3:** Sample characteristics and inferential analysis comparing the distribution of variable categories between periodontitis grade

	Periodontitis grade				
Variable	Healthy	Grade A	Grade B	Grade C	*p* -Value
Part, *N* (%)	50 (50)	3 (3)	30 (30)	17 (17)	—
Age groups					0.039 a
[18–25]	0 (0)	0 (0)	1 (3.3)	1 (5.9)	
[26–45]	39 (78)	3 (100)	8 (26.7)	11 (64.7)	
≥46	11 (22)	0 (0)	21 (70)	5 (29.4)	
Sex					0.056
Male	18 (36)	2 (66.7)	16 (53.3)	10 (58.8)	
Female	32 (64)	1 (33)	14 (46.7)	7 (41.2)	
Diabetes					0.758
No	49 (98)	3 (100)	28 (93.3)	17 (100)	
Yes	1 (2)	0 (0)	2 (6.7)	0 (0)	
Smoking					0.020 a
No	31 (62)	2 (66.7)	19 (63.3)	3 (17.6)	
Yes	19 (38)	1 (33.3)	11 (36.7)	14 (82.4)	
Alcohol					0.068
No	33 (66)	3 (100)	17 (56.7)	6 (35.3)	
Yes	17 (34)	0 (0)	13 (43.3)	11 (64.7)	
Oral hygiene					0.180
0–1 times/d	3 (6)	0 (0)	5 (16.7)	1 (11.8)	
1–2 times/d	9 (18)	1 (33.3)	6 (20)	4 (23.5)	
2–3 times/day	38 (76)	2 (66.7)	19 (63.3)	11 (64.7)	
Herpesviridae family					<0.001 a
Absence	47 (94)	2 (66.7)	11 (36.7)	7 (41.2)	
Presence	3 (6)	1 (33.3)	19 (63.3)	10 (58.8)	
Virus DNA prevalence					
HSV1					<0.001 a
Absence	49 (98)	2 (66.7)	18 (22.5)	11 (13.8)	
Presence	1 (2)	1 (33.3)	12 (40)	6 (35.3)	
HSV2					0.001 a
Absence	49 (98)	3 (100)	22 (73.3)	13 (76.5)	
Presence	1 (2)	0 (0)	8 (26.7)	4 (23.5)	
EBV					<0.001 a
Absence	49 (98)	3 (100)	23 (76.7)	8 (47.1)	
Presence	1 (2)	0 (0)	7 (23.3)	9 (52.9)	
CMV					0.019 a
Absence	50 (100)	3 (100)	25 (83.3)	16 (94.1)	
Presence	0 (0)	0 (0)	5 (16.7)	1 (5.9)	

Note: Periodontitis staging and grading 1 2 ; Percentages refer to columns.

a Significant difference.

### Prevalence of virus DNA

The prevalence of any type of Herpesviridae family virus DNA was 6% for the periodontal healthy group and 60% for the periodontitis group ( Table 1 ). The prevalence of all Herpesviridae virus DNA was significantly higher for the periodontally compromised group compared with the healthy group ( Table 1 ).

Considering the periodontitis stage, the presence of Herpesviridae family virus DNA was significant for stages II, III, and IV compared with the healthy group ( Table 2 ). Analyzing Herpesviridae-specific virus, the HSV1 DNA was prevalent in all periodontitis stages, with a higher prevalence rate in stage III (p <0.001); while HSV 2 ( *p* <0.001), EBV ( *p* <0.001) and CMV ( *p* = 0.047) DNA had increasing prevalence rates in more severe stages (stages III and IV, Table 2 ).

Considering the periodontitis grade of progression, virus DNA presence was significantly different, with an increase of Herpesviridae family virus in more severe periodontitis grades B (moderate progression) and C (rapid progression), respectively ( *p* <0001; Table 3 ). Analyzing Herpesviridae-specific virus: HSV1 viral DNA was prevalent in all grades (p <0.001), while HSV2 (p = 0.001), CMV (p = 0.019) and EBV (p <0.001) DNA were prevalent only in moderate and rapid grades of progression (grades B and C, respectively, Table 3 ). The EBV DNA registered a marked prevalence in the rapid progression grade ( Table 3 ).

## Discussion

The present study revealed a significant difference in the prevalence of DNA of Herpesviridae family virus in periodontally compromised patients. In addition, the distribution varied significantly depending on the periodontitis staging and grading, with HSV1 prevalence virtually in all stages and grades, and HSV2, EBV, and CMV more prevalent in severe stages and increased progression grades. This might suggest that different viruses may be associated with different periodontitis stages and progression grades. Previous studies registered a significant association of herpes virus (HSV-1, HSV-2, EBV, and CMV) with periodontal disease, and even to a higher standard with severe periodontal disease where EBV and CMV significantly increased in severe periodontitis, thus potentially playing a role in increasing the disease severity. 24 The association of different Herpesviridae viruses in the destruction of the periodontium registered in the present study is an important finding considering the mechanism of periodontal breakdown, analogous to previous in vitro studies. An in vitro study in gingival fibroblasts infected with Herpesviruses demonstrated a higher activity of metalloproteins I and II, alongside the reduction in collagen I and III coding mRNA expression. 30 Since periodontal tissues are essentially composed of collagen, it is deductible that these alterations lead to changes in the periodontium tissues, potentially resulting in the formation of periodontal pockets. 30 Herpesviridae virus infection is typically associated with an increase in periodontal pathogenic bacteria. Polymerase chain reaction tests revealed that both the EBV and CMV virus were associated with *Porphyromonas gingivalis, Tannerella forsythia, Prevotella intermedia, Prevotella nigrescens* , and *Treponema denticola* . Studies also demonstrated that *Porphyromonas gingivalis* can induce the production of the *zebra* protein from EBV virus, responsible for its reactivation. 31 This is particularly interesting and finds parallel in the present study, where EBV and CMV were mainly associated with more severe stages of periodontal disease (stages III and IV) at a point of periodontal destruction that either jeopardizes individual teeth or the complete dentition. Currently, numerous studies infer that an active viral infection can trigger a crisis on periodontal tissues. 8 12 15 16 17 24 28 31 32 33 34 35 36 37 38 39 40 Viruses are not seen as the main etiological factor of the disease, but as an adjuvant in its severity and clinical progression. 12

To the authors' knowledge, this is one of the first clinical and laboratorial studies to report the distribution of DNA of Herpesviridae family viruses in periodontally compromised patients using the new classification for periodontal disease. 1 2 In 2017 a new classification for periodontal disease was implemented. The past terms chronic and aggressive are now under the same category of periodontitis and are supplementary classified using a staging and grading system. The disease stages relate to the severity and complexity of the approach/treatment as well as the extent and distribution of the disease, while grades relate to the risk of rapid progression and prognosis of response to treatment. 1

There are factors that could potentially influence the study results, for example ethnicity and geography, both previously indicated as periodontal health disparities factors. A previous study investigating the prevalence and extent of periodontitis in a similar setting as the present study, registered a significant increase in disease prevalence for ethnicity in rural communities. 41 Furthermore, the global burden of oral diseases registered marked differences in the prevalence of periodontal diseases across the globe, between continents and even within the same continent. 5 Finally, age was previously reported as a factor that may imply a variation in the frequency of periodontitis. 34 The present study setting took place in a Metropolitan area, predominantly with white patients of a relatively young age which could potentially differ in characteristics (such as lifestyle). These characteristics can translate in different prevalence of Herpesviridae family virus.

The literature is not consensual in the prevalence of different Herpesviridae family virus in healthy and periodontally compromised patients. A wide range is reported across different publications, with the present study naturally positioned within the range. Various studies reported higher HSV-1 prevalence in periodontitis compared with the 38% of the present study, with 45.2, 42 76, 43 and 100%. 34 On the other hand, other studies registered lower HSV-1 prevalence: 28, 24 13, 44 4 to 13, 40 and 1.5%, 45 while reporting higher 40 or similar 45 prevalence of HSV-1 in healthy periodontium patients. A recent meta-analysis investigating the association of HSV-1 and periodontal diseases, estimated increased odds ratios of HSV-1 prevalence of 4.4 for any periodontitis (95% confidence interval, 1.9–10.2), 2.8 for chronic periodontitis (95% confidence interval, 1.0–8.3), and 11.8 for aggressive periodontitis (95% confidence interval, 5.4–25.8). 37 The results of the present study are in line with the findings of the meta-analysis, also registering a significant increase in the prevalence of HSV-1 in periodontitis patients compared with healthy patients.

Regarding HSV-2 prevalence, the literature is scarce: three studies reported 32, 24 15.8, 34 and 12% 43 in periodontitis patients (24% registered in the present study). In what concerns healthy periodontium patients, a prevalence of 4% was reported, 43 similar to 2% registered in our study.

Concerning EBV, the prevalence in periodontitis patients ranged between 3 and 45.2% 24 34 40 42 43 45 46 47 with four studies reporting comparable prevalences 24 43 46 47 to the present study (32%). For healthy patients, the prevalence of EBV ranged between 0 and 13.9% 40 42 43 45 46 ^,^ with only one report 46 registering a similar prevalence compared with the present study (2%).

The prevalence of CMV in periodontitis patients ranged between 0 and 56.2% 24 34 40 42 43 44 45 46 47 with the majority registering a higher prevalence than in the present study (12%). In healthy patients, the absence of CMV registered in the present study finds parallel in a minority of studies 45 47 whereas the majority reported a prevalence between 4.8 and 10%. 42 43 44 46 These overall differences further underline the potential effect of ethnicity and geography on the prevalence of Herpesviridae family virus in periodontium healthy and affected patients. Nevertheless, the large majority registered a higher prevalence of herpes virus in periodontitis patients when compared with healthy patients, following the same pattern of the present study.

The results of the present study stress upon the relevancy of analyzing each patient's oral microbiome, owing to the fact that even between periodontitis patients the microbiome differs from one individual to another. 48 To know a patient's periodontal microbiome and to target specific microorganisms can result in better treatment prognosis. 49 In various cases of periodontitis, the periodontal breakdown in specific sites cannot be explained by local risk factors, which raises the possibility for a co-infection of viruses and bacteria. 28 Summarily, it is important to recognize an individual/patient as the central component to a very complex disease, emphasizing the need to refine the diagnosis, treatment, and management of periodontal disease. Thus, the treatment should be tailored to fit each patient individually, and not to fit the general population, 50 and therefore the authors suggest chair-side lateral flow tests for diagnosis of Herpesviridae family viruses.

It is clear from the literature that periodontitis remains a lifelong condition which is challenging to manage from the clinical point of view for both patient and clinician. Therefore, it is important to emphasize the necessity of a robust surveillance plan for periodontal patients successfully treated. A previous 3-year epidemiological surveillance study investigating the prevalence of the main oral diseases with over 22,000 patients (including 3,497 patients with periodontitis) stressed the importance of surveillance in clinical practice. Surveillance has the objective of providing permanent technical guidance for health professionals who have the responsibility to decide on the implementation of disease control measures while using updated information on the prevalence and risk indicators in a defined population. 51 Moreover, a periodontal compromised patient remains so for life, even following successful therapy, requiring life-long supportive care to prevent recurrence of disease. 11

The strengths and limitations of the present study warrant consideration. One strength represented the type of evaluation performed, using sensitive laboratorial methods to evaluate the presence of viral DNA, together with the use of the most recent classification of periodontal disease. Importantly, this was one of the first studies to use the most recent classification of periodontal disease, providing a platform for comparability and discussion across studies. Considering the limitations, the study design, together with the preselected and small sample size (illustrated by the absence of patients with stage I periodontitis and the small sample size in stage II periodontitis) did not allow to evaluate longitudinally the true effect sizes. Moreover, it was unable to detect more distinct associations and disabled multivariable analysis, limiting the generalizability of the study results. In addition, the inclusion of patients with diabetes and smoking habits represents a limitation considering the potential to significantly affect the plaque composition. Conducting the qualitative multiplex PCR can be considered as a limitation for this study. Despite enabling the simultaneous detection of multiple targets in a single reaction and the fact that different primers were used to target the different Herpes virus, 24 qualitative multiplex PCR does not allow a more accurate estimate of the viral load when compared with other PCR methods. Consequently, future investigation using a quantitative PCR technique should be performed to estimate more accurately the role of Herpesviridae family virus in periodontitis. Future studies should examine longitudinally the influence of these viruses on the periodontal outcome, investigating the effect size of each specific viral load, its potential association with periodontitis staging and grading while controlling for other variables of interest such as smoking and diabetes.

## Conclusion

The etiopathology of periodontal disease can include active Herpesviruses present in virtually all degrees of periodontal disease staging. A significant different distribution of specific Herpesviridae virus DNA per each stage of disease was registered.
