# Supplementary material for: Association of Herpesvirus and Periodontitis: A Clinical and Laboratorial Case–Control Study
Source: Eur J Dent. 2023 Jun 9;17(4):1300–8. doi: 10.1055/s-0043-1761423 (PMC10756839; doi:10.1055/s-0043-1761423)
Supplement: Supplementary file 1 — Supplementary Material [file 10-1055-s-0043-1761423-s202292398.pdf]

# Appendix

## Methods

### Survey Variables and Periodontal Diagnosis

Socio-demographic factors such as gender, age, oral hygiene habits, alcohol consumption, and smoking patterns, as well as the presence or absence of diabetic pathology were obtained through surveys given to the patients alongside the informed consents. Gender was classified as male or female. All the surveys were numbered from 1 to 100 so they could be later matched with the samples, allowing identification. To analyze hygiene habits, tooth brushing frequency was recorded under gaps of zero to one times, one to two times, or two to three times per day. Alcohol consumption was addressed under the frequency of daily, weekly, or monthly ingestion. Smoking patterns were classified into gaps, corresponding to the number of cigarettes smoked as 1 to 9, 10 to 19, or more than 20 cigarettes per day.

All examinations to diagnose the periodontal status were conducted on an illuminated dental chair and using a periodontal probe. First, the existing teeth in mouth were counted, and the examination to obtain the values of both the gingival index and plaque index was carried. Consequently, probing depth was measured in all existing teeth, beginning in the first quadrant (most distal tooth) and finishing in the fourth quadrant (most distal tooth).

The probing depth of every existing tooth was measured (in millimeters) in six different locations: distal-buccal, distal-palatine, central-buccal, central-palatine, mesial-buccal, and mesial-palatine. While probing, bleeding from any of these six locations was also recorded. Following, the distance of the gingival margin to the cement–enamel junction was measured (in millimeters) according to those six aforementioned locations (per tooth) using a periodontal probe. Having both the probing debts and the gingival margin the clinical attachment loss (CAL) was calculated and registered. Moreover, furcation involvement was investigated and registered if present.

Every tooth in mouth was also tested for mobility and registered if pathological mobility patterns were present. Finally, periapical radiographs were taken to analyze the bone loss scheme. After the examination, the periodontal diagnosis was given under the classification of staging and grading, according to the 2017 World Periodontal workshop for the classification of Periodontal diseases.[1]

### Sample collection

For the extraction of the crevicular fluid samples, two numbered 35 paper points were used per participant. The paper points were previously sterilized in an autoclave under 134 °C and consequently placed in sterilized Eppendorfs of 1.5 mL capacity.[2] The Eppendorf were numbered from 1 to 100 for the purpose of later matching the sample number to the survey number, allowing future identification. For instance, the patient filling in the survey number 37 got his crevicular fluid collected with the paper points addressed to the Eppendorf number 37.

For the crevicular fluid sample collection the two deepest periodontal pockets (in mouth) were chosen. The sample collection was done on an illuminated dental chair. Using a periodontal probe, the selected pockets were remeasured to confirm their depth. Following, the collection site was dried with an air jet (sani-tip) so local saliva could be removed. Subsequently, the paper point was inserted in the periodontal socket and let to absorb the crevicular fluid for 30 seconds.[3] This process was repeated and done on other location of the mouth, in a periodontal socket, that was similarly, large in depth. In total two sterilized paper points were used per participant.

After the sample collection the paper points were placed in the respective sterile Eppendorf for future laboratory analysis with PCR technique.

In the healthy group the process was similar, however, the paper points were placed in the gingival sulcus of molars or premolars for the collection of crevicular fluid.

## Sample analysis

### DNA extraction

The analysis of the crevicular fluid samples took place at the Egas Moniz Applied Microbiology Laboratory.

First, DNA extraction was executed. The procedure for the DNA extraction was carried for each one of the 100 samples individually, following the same protocol. Nitril gloves were used throughout the whole process.

To each sample Eppendorf, 1 mL of ultrapure water (Sigma, Portugal) was added with a micropipette and let to rest at room temperature for 30 minutes. Subsequently, the Eppendorfs were vortex for 10 seconds at low rpm. Following, the samples were placed in a centrifuge for 3 minutes at 14,000 rpm. After being centrifugated the supernatant was removed, not damaging the pellet with a micropipette. The tip of the micropipette was discarded and a sterile one was used for every single one of the samples. Consecutively, to each sample 200 µL of Chelex (Bio-Rad, Portugal) 5% was added and then placed in bain-marie (56°C) for 15 minutes. The samples were vortexed for 10 seconds at high rpm and the tips from the caps pierced with a sterile needle. Following, the samples were placed in floating supports on boiling water for 8 minutes, not letting surpass the protocoled time. Posteriorly, the samples were vortexed (10 seconds) and centrifuged (14,000 rpm for 3 minutes), one final time. Succeeding the DNA extraction procedure, the samples were stored in a freezer at −20°C for future use.

 © 2023. The Author(s).

## Polymerase Chain Reaction

The technique used in the study was qualitative multiplex PCR. A real time thermal cycler was not available to enable the quantification of viral load. Multiplex PCR enables to simultaneously detect multiple targets in a single reaction. For the preparation of PCR solutions, reverse and forward primers were used for HSV1, HSV2, CMV, and EBV (►**Appendix Table 1**),[4] with 25 µL of MaterMix Taq Polymerase (Nzytech, Lisbon, Portugal), 14 µL of ultrapure water and 1 µL of each primer were added to numbered Eppendorfs. Subsequently, 3 µL of the previously extracted DNA sample was pipetted.[4] A control Eppendorf was prepared, containing all the aforementioned solutions, however, the 3 µL of genetic material (DNA) was replaced by ultrapure water.

Primer solutions were prepared with a concentration of 10 µM and stored at –20°C. This procedure as well as the following, was executed with proper refrigeration and nitril gloves, as per protocol.

To prepare the PCR solutions: 25 µL of *Taq Polymerase MasterMix* (Nzytech), 1 µL of each primer and 14 µL of ultrapure water (Sigma), and 3 µL of DNA were added to each Eppendorf. Alongside the preparation of each 10 PCR solutions, a control solution was prepared to ensure that no contamination occurred during the experience. The control solution was similar to the PCR ones, however, 3 µL of DNA was replaced by 3 µL of ultrapure water. The prepared PCR solutions, alongside the control solution, were placed in a thermocycler. The program used in the thermocycler consisted of 95°C for 5 minutes for initial denaturation; 45 cycles, where each cycle consists of 30 seconds at 95°C (denaturation), 30 seconds at 54°C (annealing) and 30 seconds at 72°C (extension), and the final extension at 72°C for 5 minutes.[4] The entire process in the thermal cycler lasted approximately two and a half hours.

For the preparation of the agarose gel (2%), 4 g of agarose and 200 mL of TAE (Tris-acetate-EDTA) were used. Furthermore, 10 µL of RedSafe nucleic acid staining solution dye (Merck, Algés, Portugal) was added. The agarose solution was then poured into a 22-well comb bed.

On a disinfected stand the Eppendorfs containing the PCR solutions were placed on ice and pipetted into the respective wells with a micropipette. The tips of the micropipette were always discarded between wells: moreover, the procedure was done using nitril gloves. Two DNA band markers, V and VI (Nzytech) were applied, which by having guide DNA bands on the order of 200 nanometers (nm), 300 nm and 400 nm, allowed better identification of the viruses. The base pairs of the respective virus' bands are listed in ►**Appendix Table 2**.[4] Positive control tests for the investigated viruses were not used, due to unavailable resources.

Each PCR solution was applied in the respective well with a 25 µL micropipette. The micropipettes were discarded and changed after every PCR solution placement. First, the V marker was applied; an interval well was left, and 25 µL of the control solution was poured. Subsequently, 25 µL of the samples were added in 10 sequent wells; Finally, leaving one well gap, the marker VI was applied.

A power of 50 volts for 3 hours was used for the electrophoresis procedure and the results were posteriorly observed under ultraviolet light. After the observation of the agarose gel under the UV light a copy of the shown image was printed for future analysis. Consecutively, the presence or absence of viruses was recorded on an excel table for statistical analysis.

**Appendix Table 1** Primers, forward and reverse, regarding each one of the viruses[4]

| Virus | Primer | Base pairs |
|---|---|---|
| HSV1 | Forward: 5′-CGTACCTGCGGCTCGTGAAGT-3′ | 21 |
| | Reverse: 5′-AGCAGGGTGCTCGTGTATGGGC-3′ | 22 |
| HSV2 | Forward: 5′-TGGTATCGCATGGGAGACAAT-3′ | 21 |
| | Reverse: 5′-CTCCGTCCAGTCGTTTATCTTG-3′ | 22 |
| CMV | Forward: 5′-ACGTGTTACTGGCGGAGTCG-3′ | 20 |
| | Reverse: 5′-TTGAGTGTGGCCAGACTGAG-3′ | 20 |
| EBV | Forward: 5′-AGCACTGGCCAGCTCATATC-3′ | 20 |
| | Reverse: 5′-TTGACGTCATGCCAAGGCAA-3′ | 20 |

**Appendix Table 2** Virus respective base pairs

| Virus | Band |
|---|---|
| HSV1 | 271 pb |
| HSV2 | 231 pb |
| CMV | 368 pb |
| EBV | 326 pb |

**Conflict of Interest**
None declared.

## References

1 Papapanou PN, Sanz M, Buduneli N, et al. Periodontitis: consensus report of workgroup 2 of the 2017 World Workshop on the classification of periodontal and peri-implant diseases and conditions. J Periodontol 2018;89(Suppl 1):S173–S182
2 Kubo CH, Gomes APM, Jorge AOC. Influência dos métodos de esterilização na capacidade e velocidade de absorção de diferentes marcas comerciais de cones de papel absorvente para endodontia. Rev Odontol UNESP 2013;29:113–127
3 Boström L, Bergström J, Dahlén G, Linder LE. Smoking and subgingival microflora in periodontal disease. J Clin Periodontol 2001;28(03):212–219
4 Kazi MMAG, Bharadwaj R, Bhat K, Happy D. Association of herpes viruses with mild, moderate and severe chronic periodontitis. J Clin Diagn Res 2015;9(07):DC05–DC08

 © 2023. The Author(s).
